# Timed Up and Go Test With a Cognitive Task: Correlations With Neuropsychological Measures in People With Parkinson’s Disease

**DOI:** 10.7759/cureus.10604

**Published:** 2020-09-22

**Authors:** Kübra Çekok, Turhan Kahraman, Gözde Duran, Berril Dönmez Çolakoğlu, Görsev Yener, Deniz Yerlikaya, Arzu Genç

**Affiliations:** 1 Physical Therapy, Medical Park Hospital, Izmir, TUR; 2 Physical Therapy, Graduate School of Health Sciences, Dokuz Eylül University, Izmir, TUR; 3 Physiotherapy and Rehabilitation, Izmir Katip Celebi University, Izmir, TUR; 4 Physical Medicine and Rehabilitation, Graduate School of Health Sciences, Dokuz Eylül University, Izmir, TUR; 5 Neurology, Dokuz Eylül University, Izmir, TUR; 6 Izmir Biomedicine and Genome Center, Dokuz Eylül University, Izmir, TUR; 7 Psychology, Dokuz Eylül University, Izmir, TUR; 8 Physical Therapy and Rehabilitation, Dokuz Eylül University, Izmir, TUR

**Keywords:** parkinson’s disease, neuropsychological tests, reaction time, executive function, postural balance

## Abstract

Background

The Timed Up and Go (TUG) test is a simple and widely used clinical test for the assessment of lower extremity function, balance, mobility, and fall risk in various populations. The TUG has been found as a valid and reliable measure in people with Parkinson’s disease (PD). Besides, the addition of a cognitive task to the TUG (TUG-cognitive) enhances predictive validity related to fall risk in people with PD. However, further investigation is needed about the correlations of the TUG-cognitive test with neuropsychological measures in people with PD.

Methods

Thirty-three people with PD [modified Hoehn and Yahr scale, median (min-max)=2.5 (1.0-3.0)] participated in this cross-sectional study. The TUG was administered in the traditional way and with a cognitive task (counting backward by three from any number between 20 and 100). Neuropsychological measures included the Montreal Cognitive Assessment (MoCA), Trail Making Test (TMT), and the Simple Reaction Time (SRT) test for stepping. The self-reported number of falls in the last six months was also recorded.

Results

The TUG-cognitive [13.1 (SD=8.5) seconds] was significantly longer than the TUG-traditional [12.2 (SD=8.1) seconds] (p<0.01). The TUG-cognitive significantly correlated with the MoCA [(rho=-0.712), TMT part A (TMT-A; rho=0.722), TMT part B (TMT-B; rho=0.694), SRT (rho=0.794), and number of falls (rho=0.960)] (p<0.01). The TUG-traditional also significantly correlated with the MoCA (rho=-0.682), TMT-A (rho=0.684), TMT-B (rho=0.746), SRT (rho=0.755), and number of falls (rho=0.702) (p<0.01).

Conclusion

Both the TUG-cognitive and TUG-traditional strongly correlated with neuropsychological measures; while the correlations were slightly stronger for the TUG-cognitive, the difference was not significant. The TUG-cognitive can be used in the clinical practice as a simple and more informative alternative to the TUG-traditional in people with PD.

## Introduction

Many activities in daily life require individuals to perform more than one task at the same time, which is called dual-tasking. Adequate balance, coordination, attention, and judgment are required while performing dual-tasks, which require the motor and cognitive systems to work together [1]. Most falls in older people are associated with decreased dual-task performance [2]. Dual-task performance is also adversely affected in people with Parkinson's disease (PD).

In people with PD, many walking features such as decreased swing time, walking speed, and stride length tend to deteriorate. In addition, coordination and timing problems are also frequent in people with PD [3]. Clinical walking tests create a map for detecting fall risk and can help with health promotion strategies. The Timed Up and Go (TUG) test is one of the most frequently used clinical walking tests in people with PD. The TUG includes many activities that are common in daily life, such as sit-to-stand, walking, and turning. The TUG has many advantages and is commonly used in clinical and research practice related to PD [4].

To increase the TUG’s precision, additional tasks such as motor and cognitive tasks can be added to the traditional TUG test. Lundin-Olsson et al. showed that the TUG with a cognitive task (TUG-cognitive) (counting back by three) was more sensitive and specific in predicting the rate of falls in older people [5]. Many people with PD have cognitive dysfunction, which affects walking and balance and thereby increasing fall risk [6]. Therefore, it is suggested that functional motor skills should be evaluated under a variety of dual-task conditions such as in the TUG-cognitive in people with PD [7]. However, research examining the correlation between the TUG-cognitive and neuropsychological measures in people with PD is scarce. Therefore, this study aimed to investigate the correlations of the TUG-cognitive test with the neuropsychological measures by comparing it with the TUG-traditional in people with PD. Due to the nature of the dual-task condition, we hypothesized that the TUG-cognitive would have greater correlations with neuropsychological measures compared to the TUG-traditional.

## Materials and methods

Design, setting, and participants

This cross-sectional study involved a one-time assessment of people with PD. The demographic information (age, gender, education duration, and body mass index) was gathered using face-to-face interviews. The self-reported number of falls in the last six months was also recorded. The modified Hoehn and Yahr scale and the Unified Parkinson’s Disease Rating Scale (UPDRS) were applied to assess disability and symptom severity, respectively. The Hoehn and Yahr scale is used to evaluate disability and impairment related to clinical disease progression of PD. The scale originally included stages 1 through 5 [8]. The Hoehn and Yahr scale was modified with the addition of stages 1.5 and 2.5 to account for the intermediate course of PD. The stages range from 1 (unilateral involvement only) to 5 (wheelchair-bound or bedridden unless aided). The UPDRS includes series of ratings for typical symptoms of PD that are assessed in different parts: part 1 (mental dysfunction and mood), part 2 (activities of daily living), part 3 (motor section), and part 4 (treatment-related complications). Higher scores in the UPDRS indicate higher symptom severity [9].

We recruited 33 people with idiopathic PD from the Movement Disorders Clinic of Dokuz Eylül University, Izmir, Turkey. The inclusion criteria were as follows: people living in the community, ability to walk unaided for 10 m, and having modified Hoehn and Yahr stages 1-3. People with any medical conditions that would preclude or interfere with the TUG assessments (e.g., physician-diagnosed dementia, acute or terminal illness, progressive neurodegenerative diseases other than PD, major psychiatric illnesses, visual impairments that could not be corrected, and deep brain stimulation) were excluded from the study [10].

In a previous study, a strong correlation (rho ≈ 0.7) was observed between the Montreal Cognitive Assessment (MoCA) and the TUG-cognitive in people with PD [11]. The required sample size was calculated as 11 for alpha error probability=0.05 and power=0.80 using G*Power (version 3.1.9.2, Dusseldorf University, Germany). Since the number was relatively small, we decided to increase the number by three times (i.e., 33 participants).

Ethical approval

The ethical approval for this study was obtained from the Noninvasive Research Ethics Board of Dokuz Eylül University (2018/26-10). The participants provided written consent prior to their participation in the study. 

Outcome measures

Neuropsychological Evaluation

The MoCA was used to assess global cognition. The MoCA is a screening tool for mild cognitive impairment and assesses a variety of cognitive domains including attention, memory, executive function, visuospatial ability, and language. The MoCA had adequate psychometric properties in people with PD [12]. The scores on the MoCA ranged from 0 to 30 and higher scores indicated better cognitive function. The Turkish version of the MoCA also showed acceptable psychometric properties in people with PD [13].

Executive function was assessed by the Trail Making Test (TMT), which is a well-established neuropsychological screening tool with high validity and reliability [14]. The TMT has two parts. Part A (TMT-A) is a visual-scanning task and part B (TMT-B) adds to it a measure of cognitive flexibility. In the TMT-A, the participants were asked to draw lines sequentially connecting consecutively numbered circles from 1 to 25, which were randomly arranged on a page as quickly as possible. In the TMT-B, the participants were asked to connect the same number of circles in an alternating sequence of numbers and letters. The standardized Turkish version of the TMT was also available and it showed good psychometric properties [14].

The Multi-Operational Apparatus for Reaction Time (MOART; Lafayette Instrument Company, Lafayette, IN) was used with food-pads to carry out the Simple Reaction Time (SRT) test for stepping [15]. A number of practice trials were administered for familiarization with the procedure [15]. Participants performed five stepping trials and the reaction time (time taken from receipt of the stimulus to lifting the stepping foot off of the foot-pad) was recorded for each trial as milliseconds [15]. There were 10-second rest intervals between the trials [15]. The SRS score was computed as the sum of five trials [15].

TUG Assessments

The TUG-traditional and TUG-cognitive were administered in random order for each patient. Each test was repeated three times and the average of the duration was used for data analysis. The TUG-traditional test was applied by making the participants perform the following tasks: standing from a chair, walking 3 m, turning around, returning to the chair, and sitting down. Although there are many different dual-task conditions for the TUG, such as delayed memory task, digit span forward, and serial subtraction [11,16], digit span-backward is one of the most commonly used version in clinical practice [17]. Therefore, in the current study, the TUG-cognitive was applied while counting backward by three from any number between 20 and 100 [17].

Statistical analysis

Data were analyzed using IBM SPSS Statistics for Windows version 24.0 (IBM Corp., Armonk, NY). Descriptive statistics were used to describe participants’ demographic characteristics and assessment results. Checking of the normal distribution of the data was applied using the Shapiro-Wilk test and investigation of histogram graphics. Correlations between the study variables were assessed using Spearman's rank-order correlation analysis. The correlation coefficients were interpreted as strong (>0.5), moderate (0.3-0.5), and weak (0.2-0.3) [18]. The significance of the difference between the two correlation coefficients was calculated using the Fisher r-to-z transformation. Statistical significance was defined at a p-value of <0.05.

## Results

There were 33 participants; the mean age of the participants was 67.9 (SD=7.9) years, and 73% of them were male. The UPDRS score was 35.4 (SD=15.6) and the median of the modified Hoehn and Yahr stage was 2.5 (min-max=1.0-3.0). Seventeen participants (51.5%) had a fall history within the last six months and the median number of falls was 1. Table 1 presents the demographic characteristics and outcome measure results of the participants.

**Table 1:** Demographic characteristics and outcome measure results of the participants (N=33) UPDRS: Unified Parkinson's Disease Rating Scale; MoCA: Montreal Cognitive Assessment; TMT: Trail Making Test; SRT: Simple Reaction Time; TUG: Timed Up and Go test; SD: standard deviation

Variables	Values
Age in years, mean (SD)	67.9 (7.9)
Gender, n (%)	
Female	9 (27)
Male	24 (73)
Body mass index, kg/m^2^, mean (SD)	27.0 (3.3)
Education duration, years, mean (SD)	10.1 (3.8)
Number of falls in the last 6 months, median (min-max)	1 (0–4)
UPDRS, mean (SD)	35.4 (15.6)
Modified Hoehn and Yahr scale, median (min-max)	2.5 (1.0–3.0)
MoCA, mean (SD)	24.8 (2.3)
TMT-A, seconds, mean (SD)	69.0 (42.3)
TMT-B, seconds, mean (SD)	124.9 (58.4)
SRT, milliseconds, mean (SD)	712.9 (376.6)
TUG-traditional, seconds, mean (SD)	12.2 (8.1)
TUG-cognitive, seconds, mean (SD)	13.1 (8.5)

The TUG-cognitive [13.1 (SD=8.5) seconds] was significantly longer than the TUG-traditional [12.2 (SD=8.1) seconds] (p<0.01). The TUG-cognitive and TUG-traditional strongly correlated with each other (rho=0.941, p<0.01). The TUG-cognitive significantly correlated with the MoCA (rho=-0.712), TMT-A (rho=0.722), TMT-B (rho=0.694), SRT (rho=0.794), and number of falls in the last six months (rho=0.696) (p<0.01). The TUG-traditional also significantly correlated with the MoCA (rho=-0.682), TMT-A (rho=0.684), TMT-B (rho=0.746), SRT (rho=0.755), and number of falls in the last six months (rho=0.702) (p<0.01). None of the correlation coefficients was significantly different between the TUG-traditional and TUG-cognitive (p>0.05). Table 2 presents the correlations between TUG tests and neuropsychological measures.

**Table 2:** Correlations between TUG tests and neuropsychological measures in people with PD (N=33) *p<0.05; ^a^Significance of the difference between the two correlation coefficients PD: Parkinson’s disease; TUG: Timed Up and Go test; MoCA: Montreal Cognitive Assessment; TMT: Trail Making Test; SRT: Simple Reaction Time; (-): not reported

	TUG-cognitive		TUG-traditional		
Variable	rho	P-value	rho	P-value	P-value^a^
TUG-cognitive	-	-	0.941	<0.001*	-
SRT	0.794	<0.001*	0.755	<0.001*	0.703
TMT-A	0.722	<0.001*	0.684	<0.001*	0.771
TMT-B	0.694	<0.001*	0.746	<0.001*	0.675
MoCA	-0.712	<0.001*	-0.682	<0.001*	0.818
Number of falls in the last 6 months	0.696	<0.001*	0.702	<0.001*	0.960

## Discussion

In the current study, we have shown that both the TUG-traditional and TUG-cognitive correlated with different neuropsychological measures and the number of falls in the people with PD. Moreover, the TUG-cognitive had a slightly greater correlation coefficient with the cognitive function, visual attention and task switching, and reaction time compared to the TUG-traditional. However, there was no significant difference between the correlation coefficients.

The completion time of the TUG-cognitive was significantly longer than that of the TUG-traditional in people with PD. In the walking tests, cognitive and motor regions of the brain work together. As the second task is assigned in the TUG-cognitive test, the process becomes complicated and the walking speed is affected. This result can be interpreted according to the capacity theory [19]. When two tasks are performed at the same time, competition within the limited resources causes deterioration in the performance of the single or both tasks. Walking assessment with a cognitive task requiring cognitive flexibility is important since cognitive flexibility is an early cognitive impairment in people with PD [20]. The current study has demonstrated that the TUG-cognitive test had more completion time compared to the TUG-traditional test. Therefore, the TUG-cognitive could be chosen as a simple clinical test that can show impairment in the dual-task performance and cognitive function in the people with PD.

Our study also showed that TUG-cognitive significantly correlated with the cognitive function as assessed by the MoCA, which is an internationally accepted tool that is frequently used in clinical and research settings [21]. However, it has been emphasized in some studies that the limitation of the MoCA in evaluating executive function and attention should be taken into consideration [22]. Therefore, we also investigated the correlations of visual attention and task switching, as well as reaction time for stepping. We demonstrated that TUG-cognitive significantly correlated with cognitive function, visual attention and task switching, and reaction time. In people with PD, early attention and executive dysfunctions result from basal ganglion pathology, and they have difficulty in appropriately performing a dual-task of cognitive performance. Therefore, it is inevitable that dual-task performance and executive function and attention should be related [23]. They are effective components of executive functions on will, self-awareness, planning, focus, monitoring response, and walking attention [24]. Impairment of one or more of the executive function components affects a person's ability to walk safely and effectively [25]. Therefore, impaired executive function leads to difficulties in dual-task conditions and, consequently, increases fall risk.

It has been shown that prolonged reaction time is an independent risk factor for falls in people with PD [26]. Intact cognitive function is important for fall avoidance by providing safe foot placement and may elucidate why reduced cognitive performance has been strongly associated with falls in people with PD [27]. Therefore, simple clinical assessment methods that significantly correlate with the above-mentioned parameters are important to quickly evaluate people with PD and to consult them for further detailed assessments. Both the TUG-traditional and TUG-cognitive significantly correlated with several important neuropsychological aspects including reaction time for stepping and number of falls within the last six months. Although there was no significant difference between the correlation coefficients, the TUG-cognitive had slightly greater correlation coefficients compared to the TUG-traditional. In addition, the completion time of the TUG-cognitive was significantly longer than that of the TUG-traditional. We speculate that TUG-cognitive would have significantly greater correlations compared to the TUG-traditional in people with PD having more disease severity, which should be investigated in future studies. In addition, due to the mild disability levels of the participants, counting backward by three as a cognitive task could be easy for the participants. A more complex attention activity such as a serial subtraction test would be better in such populations [16]. In future studies, different protocols for cognitive demand in the TUG should be investigated. Nevertheless, the TUG-cognitive can be used in clinical practice as a simple and more informative alternative to the TUG-traditional in people with PD.

Dual-task performance is affected by many factors such as balance, cognitive performance, cognitive load, walking ability, and environmental factors [28]. Therefore, psychometrically sound assessment methods are highly required. In this study, we have shown significant correlations of TUG-cognitive with several neuropsychological assessments and the number of falls, which supports its validity in people with PD.

Limitations

This study has some limitations. Firstly, there was the absence of a control group, and having a healthy control group would be better to demonstrate whether the observed correlations were specific to people with PD. Secondly, the participants had mild to moderate disease severity, which limits the generalizability of our results. Finally, the current study supports that the TUG-cognitive has robust construct validity against neuropsychological measures in people with PD. However, the different important psychometric properties of the TUG-cognitive such as test-retest and interrater reliability, discriminant validity, and minimal clinically important difference should be investigated in future studies.

## Conclusions

Both the TUG-cognitive and TUG-traditional strongly correlated with the neuropsychological measures and the number of falls, yet the TUG-cognitive had slightly greater associations with some of the neuropsychological measures. People with PD showed significantly less performance levels in the TUG-cognitive compared to the TUG-traditional. The TUG-cognitive can be used in clinical practice as a simple and more informative alternative to the TUG-traditional in people with PD. However, we speculate that the TUG-cognitive would have significantly greater correlations compared to the TUG-traditional in PD patients with higher disease severity, which should be investigated in future studies.
